# Robotic-assisted partial nephrectomy: Has it come of age?

**DOI:** 10.4103/0970-1591.57929

**Published:** 2009

**Authors:** Manish N. Patel, Mahendra Bhandari, Mani Menon, Craig G. Rogers

**Affiliations:** Vattikuti Urology Institute, Henry Ford Hospital, Detroit, MI, USA

**Keywords:** Kidney cancer, partial nephrectomy, robotics

## Abstract

Surgical resection is the gold standard for the treatment of renal cell carcinoma, and partial nephrectomy (PN) is the treatment of choice for tumors smaller than 4 cm in size. A laparoscopic PN is a viable alternative to a traditional open PN, demonstrating good oncologic and functional outcomes. A laparoscopic PN is a challenging procedure, particularly performing intracorporeal suturing under the time constraints of warm ischemia. The introduction of the da Vinci surgical system (Intuitive Surgical Inc., Sunnyvale, CA) with wristed instruments and magnified, 3-dimensional vision may facilitate the technical challenges of a minimally invasive PN. The technique of robotic partial nephrectomy (RPN) is still evolving and a number of institutions have recently reported their results. In this article, we present a review of the literature and our technique for robotic PN using a transperitoneal approach.

## INTRODUCTION

Due to the increased use of cross-sectional imaging, the number of small renal masses being detected is rising. Surgical resection is the gold standard for treatment of renal cell carcinoma, and partial nephrectomy (PN) is the treatment of choice for tumors smaller than 4 cm in size.[1] However, PNs are underutilized and many patients are receiving radical nephrectomies (RN).[2] An open PN has been show to have equivalent cancer control as compared with a RN with the obvious advantage of preserving renal function.[3,4] A laparoscopic PN is a viable alternative to a traditional open PN as it has been shown to achieve long-term cancer remission and renal function results.[5–8] A laparoscopic PN, however, is technically challenging and requires specialized training and experience to perform a tumor resection and renal reconstruction within the time constraints of warm ischemia.

The introduction of the da Vinci surgical system (Intuitive Surgical Inc., Sunnyvale, CA) with wristed instruments and magnified, 3-dimensional vision may facilitate some of the technical challenges during laparoscopy including intracoporial suturing and renal reconstruction. The feasibility of robotic partial nephrectomy (RPN) has been demonstrated in a number of series demonstrating similar perioperative outcomes such as warm ischemia time, blood loss, length of stay, and OR time.[9–12] These early reports demonstrated acceptable positive margin rates, warm ischemia time, and perioperative outcomes in a small, relatively exophytic tumor. More recent reports have demonstrated the feasibility of performing RPN for more complex tumors including endophyitic, hilar, and multiple tumors.[13,14] Rogers, *et al*. has published the largest series of RPN with 148 patients from 7 centers undergoing RPN.[15] In this series, RPN results appear comparable to open PN, making RPN a feasible option for patients wishing to undergo a minimally invasive nephron sparing surgery.

RPN is still in its infancy compared with laparoscopy. The largest single center comparison of the techniques was published by Wang, *et al*. comparing RPN and LPN in 100 consecutive patients demonstrating a lower mean warm ischemia time, blood loss, and length of stay with RPN.[16] The clinical significance of decreased blood loss and length of stay are debatable, but the reduction in mean warm ischemia time of 8 minutes using the sliding hemolock clip technique is likely beneficial.

The technique of RPN can be learned by many surgeons as Deane, *et al*. have demonstrated; a fellowship-trained surgeon experienced in open PNs and robotic prostatectomy can perform a RPN with operative parameters and outcomes similar to experienced laparoscopic surgeons performing laparoscopic PNs.[17]

In this article, we present our technique for robotic PN using a transperitoneal approach.

## PLANNING AND PREPARATION

### Indications and patient selection

Indications for PN have been published[1] and include routine performances in patients with an anatomic or functional solitary kidney, or evidence of tumor in the contralateral kidney. A PN can be performed electively in patients with localized renal cell cancer (RCC) and a normally functioning contralateral kidney. For tumors smaller than 4 cm, recurrence rates are similar to those for a RN,[1] thus a PN is generally performed. For select patients however, a PN can be performed for larger masses.[18] Patients with complex tumors (hilar, endophytic, or multiple) are also candidates for a PN; however, these surgeries are advanced procedures and should be done on select patients by a surgeon with considerable experience. If the patient does not meet these criteria, a RN is recommended.

A minimally invasive approach to PN can be used for almost any patient undergoing consideration for this procedure. Relative contraindications to a minimally invasive approach include extensive prior abdominal surgery and patients with renal insufficiency who cannot tolerate the demands of warm ischemia.

### Patient specific preparation

All patients being considered for RPN undergo a metastatic workup including imaging with an abdominal computed tomography (CT) scan or magnetic resonance imaging (MRI), an Anterior-Posterior, and a lateral chest X-ray. Additional imaging such as a chest CT, head CT, and bone scan are ordered based on clinical signs and symptoms of metastasis. In addition, all patients have blood work done including electrolytes, creatinine, BUN, a complete blood count, coagulation studies, and liver function tests.

All patients are instructed to stop any anticoagulants at least 5 days before surgery. Patients are given a bowel prep of magnesium citrate and are instructed to take it the day before surgery. They are also instructed not to eat or drink anything after midnight the night before surgery. A first generation cephalosporin is routinely given perioperatively about 30 minutes prior to incision.

### Surgical team

The surgical team includes 1 operating console surgeon, 1 bedside assistant, and 1 surgical technician. The surgical technician prepares the back table followed by draping the robot. For an experienced technician, draping the robot takes about 5 minutes. The operating surgeon may scrub initially to assist in patient preparation and port placement and then breaks scrub prior to sitting at the robotic console. The bedside team remains scrubbed throughout the case and assists the console surgeon during the procedure.

### Specific equipment/materials

- Instruments used by the console surgeon
  Tumor identification and excision
  - Right hand - hot shears, monopolar curved scissors, or monopolar hook
  - Left hand - Maryland bipolar forceps, prograsp forceps, or fenestrated bipolar forceps
  Renal reconstruction
  - 2 large needle drivers or 1 needle driver in the right hand and forceps in the left hand
- Fourth robotic arm instruments (optional)
  - Dual blade retractor
  - Double fenestrated retractor
- Instruments used by the bedside assistant
  - 5 mm laparoscopic grasper
  - 5 mm laparoscopic needle driver
  - 5 mm laparoscopic scissors
  - 5 mm and 10 mm hemolock clip applier
  - 10 mm specimen extraction bag
  - Laparoscopic bulldog clamp applier
  - Suction tip and irrigator
  - Jackson-Pratt drain

### Patient positioning

General endotracheal anesthesia is used for this procedure. A Foley catheter is placed before positioning the patient. The patient is placed in the full flank position. Mild table flexion is used to increase the space for ports with the kidney placed in the center of the table break. The arms are padded at the elbows, wrists, and hands and are extended in front of the patient with the upper arm suspended. The lower leg is flexed, the upper leg is straight, and all lower extremity pressure points are padded. The patient is secured to the table at the chest, iliac crest, and knees with a wide cloth tape and velcro straps to ensure the patient does not move during the procedure. Pressure points are inspected and additional padding is placed if necessary.

## SURGICAL STEPS

### Step 1: Trocar placement

Placing ports for robotic surgery is similar to laparoscopy and takes approximately the same amount of time. A 12 mm port for the da Vinci camera can be placed laterally using a 0° or 30° angle up scope[11,19] or medially using a 0° or 30° down scope.[14] With a medial camera position, two 8 mm da Vinci ports are placed under vision approximately 5–6 cm away from the camera. These three ports are triangulated towards the renal hilum. With a lateral camera position, the ports are in a perpendicular line to the line drawn from the camera port to the hilum. A port for the fourth robotic arm may be placed approximately 4–5 fingerbreadths medially to the most caudal robotic instrument port. A 12 mm assistant port is placed near the umbilicus or lateral to the rectus in obese patients. An optional 5 mm assistant port can be placed below the 12 mm port, if necessary. For right sided cases, a 5 mm subxiphoid port can be placed for retraction. The robot is docked posteriorly at approximately a 20° angle towards the head of the patient.

### Step 2: Medial mobilization of the bowel

The colon must be mobilized medially to expose the kidney. The white line of Toldt is incised lateral to the colon by cutting the superficial layer of the peritoneum. The colon is retracted medially by the assistant while the relatively avascular plane between the posterior mesocolon and anterior Gerota's fascia is developed. This dissection is continued to the upper pole of the kidney. The perinephric fat under Gerota's can be distinguished from mesenteric fat by its paler yellow color. This difference in color may help orient the surgeon if the dissection leaves the desired plane. Care is taken not to use cautery near the colon to avoid a thermal injury.

### Step 3: Identify anatomical landmarks

A plane is developed between the packet containing the ureter and gonadal vein and the psoas muscle. Gerota's fascia is grasped and the kidney is lifted anteriorly to help expose the ureter and gonadal vein. The fourth robotic arm can be used to help lift the kidney and ureter to facilitate dissection. The ureter and gonadal vein are identified and traced to the renal hilum.

### Step 4: Hilar dissection

The hilum is identified by tracing the gonadal vein superiorly. On the left, the gonadal is traced to its insertion in the renal vein. On the right, the gonadal is traced to the vena cava, then followed to the renal vein. Once the renal vein is identified, the renal artery is dissected. The artery usually sits behind the vein, and visualization of arterial pulsations may aid in identifying its exact location. The hilar vessels are dissected and small venous branches and lymphatics are divided. Dissection may be facilitated by using the fourth robotic arm to retract the kidney laterally placing the renal hilum on stretch.

### Step 5: Tumor identification

**Figure 1:**
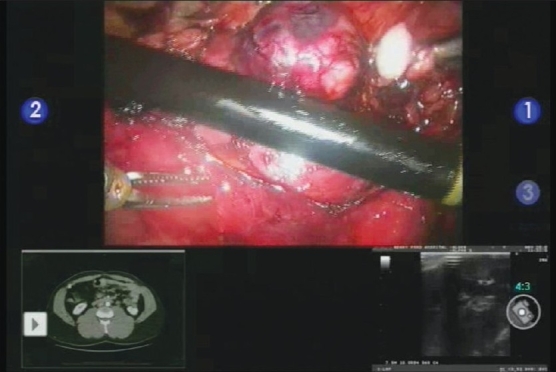
Use of TilePro during a robotic partial nephrectomy. TilePro image demonstrating the ability to simultaneously view live intraoperative ultrasound image (right lower corner) and preoperative CT image (left lower corner) on the console screen during a robotic partial nephrectomy. TilePro is being used to delineate tumor margins prior to tumor excision

A flexible laparoscopic ultrasound is introduced through the 12 mm assistant port. Intraoperative ultrasound images and preoperative radiographic imaging can be displayed on the console screen as a picture on picture display using the TilePro feature of the da Vinci S system [Figure 1]. The tumor is identified and Gerota's fascia is opened over the tumor. The perinephric fat over the tumor is removed and sent to pathology as a frozen section. Adequate fat is removed to expose normal parenchyma on all sides of the tumor to facilitate future capsular reconstruction. The ultrasound probe is again used to demarcate the tumor margins and depth. Cautery is used to score the renal capsule demarking the planes of resection.

### Step 6: Hilar clamping

Prior to clamping, ensure that all stitches and instruments are available for resection and renal reconstruction in order to minimize warm ischemia time. Ensure there is adequate CO2 for insufflation and that the patient has received 12.5g of manitol for osmotic diuresis.

There are two commonly used methods for hilar clamping, laparoscopic bulldog clamps and a Statinsky clamp. Laparoscopic bulldog clamps are placed by the assistant through the 12 mm port. The artery is test clamped to ensure the entire vessel can be occluded. The renal artery is clamped first, followed by the renal vein. If the tumor is small or exophytic, the renal artery alone may be clamped.

If individual dissection of the renal vessels is difficult, en bloc clamping of the hilum can be performed with a Statinsky clamp. This clamp should be placed parallel to the aorta and inferior vena cava. Use of the Statinsky clamp requires a dedicated port, therefore the assistant must be able to perform all tasks through a single port or an additional port must be placed. Care must be taken to avoid movement of the clamp or collision with any of the robotic arms as this may cause injury to the renal vessels.

### Step 7: Tumor excision

**Figure 2:**
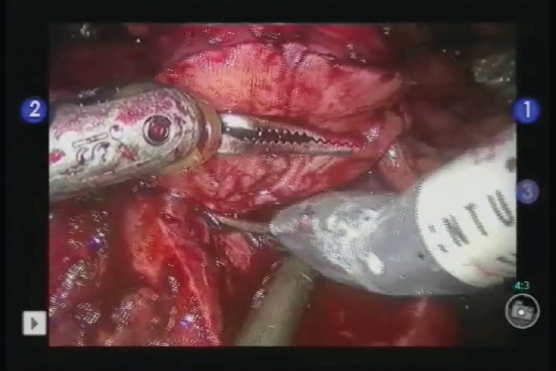
Excision of the tumor during a robotic partial nephrectomy. Excision is preformed using cold Monopolar scissors in the right hand and a Maryland bipolar in the left hand. The left hand is turned 90° to provide upward traction while the assistant uses the suction to provide downward traction

Cold excision with monopolar scissors is used along the demarcated margins to remove the tumor [Figure 2]. As curing the cancer is the primary concern, a small margin of normal parenchyma is also excised. If the tumor is entered, the scissors are backed up and the plane of excision is corrected. A ureteral catheter can be placed prior to surgery; however, we do not routinely place them as the collecting system can be visualized sufficiently with the improved magnification of the robotic camera. During excision, the assistant uses a suction tip to clear any blood in the surgical field as well as apply counter traction on the renal parenchyma to help delineate the plane of resection. Once the tumor is removed, it is placed out of the way for later retrieval once the clamps have been removed.

### Step 8: Renal reconstruction

The robotic instruments are replaced with needle drivers. We currently prefer a needle driver in the right hand and Prograsp forceps in the left hand. Prograsp forceps allow for effective grasping of needles and sutures and can throw stitches if the right arm is occupied.

#### a) Inner layer closure

A 3-0 or 4-0 vicryl on a RB-1 or SH needle is used to repair any entry into the collecting system and achieve hemostasis. A running baseball stitch starting at the far end of the defect and working towards the camera is performed. The end of the suture is prepared with a preplaced Lapra-Ty to avoid knot tying, and another Lapra-Ty is placed by the assistant to secure the stitch when the suturing is completed. If bleeding continues, additional stitches are placed as needed.

#### b) Capsule reconstruction

**Figure 3:**
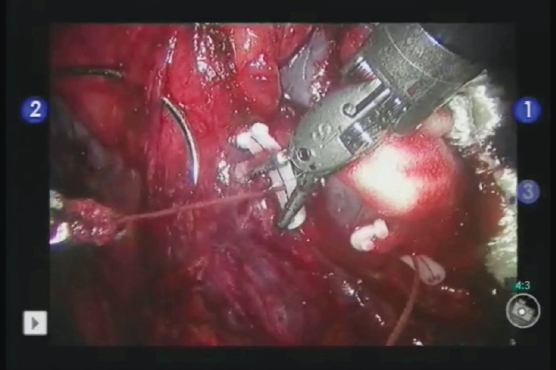
Capsular closure using the sliding hemolock clip technique. A 0-vicryl suture is placed through the capsule on each side to compress the defect. A hemolock clip is placed on the near side, and the surgeon using the robotic instrument slides the clip down to cinch the defect closed

0-vicryl sutures on a CT-1 needle cut to a length of 5 inches are prepared with a hemolock clip on the outer end secured with a Lapra-Ty and knot. Interrupted stitches are placed to help reapproximate the capsular edges starting at the far side of the defect and working toward the camera. Large bites of the capsule are taken to ensure the suture does not rip through. The stitches are secured with hemolock clips which are slid down the suture by the console surgeon [Figure 3].[20] The interrupted stitches with a hemolock clip spreads the force of the suture over a larger surface area allowing stitches to be cinched tighter for a closer reapproximation of the edges and better homeostasis. This robotic technique allows for better reapproximation of capsular edges versus laparoscopy as the console surgeon is able to slide the hemolock clip down the suture for appropriate tension, a maneuver not available to laparoscopic surgeons. If the defect is large, Surgicel bolsters can be positioned under the sutures with a homeostatic agent such as Floseal. After clamp removal, Lapra-Ty clips are placed by the assistant on the capsular stitches next to the hemolock clips to secure them. The sutures are cut and the needles are removed.

### Step 9: Removal of Hilar clamps

Following reconstruction of the renal defect, the Hilar clamps are removed. The venous clamp is removed first followed by flashing of the arterial clamp to confirm homeostasis. Homeostasis can also be tested by reducing the pneumoperitoneum to 5 mmHg. If oozing continues, pressure can be applied by a laparoscopic sponge. Another 12.5g of manitol is given once the clamps are removed.

### Step 10: Specimen Retrieval and Closure

The specimen is retrieved from the pelvis and placed into an extraction bag inserted through the 12 mm assistant port. A Jackson-Pratt drain may be placed through the fourth robotic arm port and is secured with a nylon suture. The 12mm assistant port incision is extended to remove the tumor specimen. The fascia of the incision is closed with interrupted 0- braided polyester sutures. The skin is closed with 4-0 braided polyglactin, subcuticular sutures, and sterile strips.

## POST-OPERATIVE CARE

A complete blood count, creatinine, and BUN are ordered in the recovery room and 12 hours post-operatively. Overnight, patients receive IV fluids, analgesics as necessary, prophylaxis for deep vein thrombosis with subcutaneous heparin, and antibiotic prophylaxis per the hospital protocol. For the first 12 to 24 hours post-operatively, patients are not given a diet and on bed rest to minimize the risk of bleeding. The morning following surgery, the Foley catheter is removed, a clear liquid diet is started, and patients are encouraged to ambulate. Patients usually remain in the hospital for 2 days.

## CONTROLLING BLEEDING DURING SURGERY

During a laparoscopic PN, hemorrhage occurs in 3.5% of patients intraoperatively.[21] Bleeding can be from a number of sources and various techniques can be used to control it. Bleeding during dissection of the hilum or ureter/gonadal can generally be controlled using a lap pad or cottonoid to tamponade the bleeding while working on a different area for a few minutes. If a bleeding vessel is identified, robotic forceps or a locking grasper can be used to occlude the vessel until cautery or clips control the bleeding.

Bleeding while on clamp can occur due to a number of reasons:[1] the main renal artery has a branch that was missed during clamping or[2] an accessory artery, usually from the adrenal, is still perfusing the kidney. Placing a long bulldog across the renal hilum to encompass all branches of the artery and another in the fat between the kidney and the adrenal to occlude any accessory arteries may help. In addition, if the renal vein is clamped, this clamp may be removed to help alleviate renal congestion and improve vision.[3] Old bulldog clamps may have decreased clamping force allowing flow to enter the kidney. In this case a second bulldog is placed distal to the original clamp. If time is needed to determine the source of bleeding, the pneumoperitoneum can temporally be increased to reduce bleeding.

## COSTS

A detailed cost-benefit analysis is beyond the scope of this paper, which emphasizes surgical technique. However, we believe there are potential advantages of using robotic assistance for PN to achieve a nephron-sparing and minimally invasive approach that may justify the costs for select patients with more complex tumors. The main costs of robotics are in the initial purchase and setup of the robot itself. While this may be prohibitive for some institutions, those that already have a robot may achieve an economy of scale by utilizing robotic assistance to a greater degree by performing renal surgery in addition to traditional prostate surgery. While we do not necessarily advocate a robotic approach for all kidney surgeries, we feel that robotic assistance may help some patients to receive a nephron sparing and minimally invasive approach who might otherwise undergo open PN or RN, thus potentially justifying increased costs for these specific patients.

## CONCLUSIONS

Laparoscopic PN is a proven technique that has been shown to provide comparable long-term functional and cancer control rates to RN for select patients. However, a laparoscopic PN is a challenging procedure requiring advanced laparoscopic skills. A RPN may facilitate some of the technical challenges of laparoscopy potentially allowing more patients to undergo a minimally invasive, nephron sparing surgery who would otherwise undergo a total nephrectomy or open surgery. The incorporation of wristed instruments, magnified 3-dimensional vision, and a tremor free platform allows easy transition for conventional open surgeons.
